# In Vitro Micropropagation of Native Ulluco (*Ullucus tuberosus* Caldas) from the Amazonas Region of Peru

**DOI:** 10.3390/plants15060959

**Published:** 2026-03-20

**Authors:** Deyli Mailita Fernández-Poquioma, Erika Llaja-Zuta, Angel David Hernández-Amasifuen, Jorge Alberto Condori-Apfata

**Affiliations:** Instituto de Investigación, Innovación y Desarrollo para el Sector Agrario y Agroindustrial (IIDAA), Facultad de Ingeniería y Ciencias Agrarias, Universidad Nacional Toribio Rodríguez de Mendoza de Amazonas, Calle Higos Urco 342—Ciudad Universitaria, Chachapoyas 01000, Peru; 7223347721@untrm.edu.pe (D.M.F.-P.); 7262322721@untrm.edu.pe (E.L.-Z.); angel.hernandez@untrm.edu.pe (A.D.H.-A.)

**Keywords:** in vitro conservation, germplasm, nodal segments, ex vitro acclimatization, plant tissue culture

## Abstract

Ulluco (*Ullucus tuberosus* Caldas) is an Andean tuber crop of high nutritional and genetic importance. However, its vegetative propagation promotes the accumulation of pathogens and limits the availability of uniform, high-quality planting material. In this study, an efficient and reproducible in vitro micropropagation protocol was established for an ulluco genotype from the Amazonas region of Peru. Nodal segments were cultured on MS (Murashige and Skoog) medium supplemented with 6-benzylaminopurine (BAP) or kinetin (KIN) at increasing concentrations (0.0–2.0 mg L^−1^). For rooting, in vitro-derived shoots were transferred to MS medium supplemented with indole-3-butyric acid (IBA) or 1-naphthaleneacetic acid (NAA) at the same concentration range (0.0–2.0 mg L^−1^). The explants exhibited a high basal morphogenetic capacity; however, the addition of cytokinins significantly enhanced the response. KIN at 2.0 mg L^−1^ achieved 100% regeneration, whereas BAP at 0.2 mg L^−1^ maximized shoot proliferation, producing 2.07 shoots per explant. Shoot elongation was greater with KIN at 1.0 mg L^−1^, reaching 39.15 mm. In the rooting phase, the response varied depending on the type and concentration of auxin. NAA at 0.1 mg L^−1^ resulted in 100% rooting and produced the greatest root length (41.93 mm), whereas IBA at 0.1 mg L^−1^ maximized the number of roots (4.67), although roots were shorter. Rooted plantlets exhibited 100% survival after eight weeks of acclimatization. This protocol provides an effective system for the rapid production of vigorous and uniform clonal plants and represents a useful tool for the propagation, conservation, and future biotechnological improvement of ulluco.

## 1. Introduction

Ulluco (*Ullucus tuberosus* Caldas) is an Andean tuber belonging to the family Basellaceae, cultivated primarily in the high Andean regions of Peru, Bolivia, Ecuador, and Colombia, where it serves as an essential food crop due to its high nutritional value and its contribution to rural food security [1,2]. Beyond its role as a food source, ulluco is also recognized as a plant genetic resource because of its remarkable morphological and genetic variability, resulting from a long process of domestication and adaptation to different altitudinal environments [3,4]. This diversity provides a strategic foundation for conservation and breeding programs; however, it also requires efficient tools to enable the multiplication, preservation, and traceability of valuable genotypes [5,6].

However, ulluco cultivation faces several constraints that limit productivity and the safe exchange of planting material. Vegetative propagation through tubers, a common practice in traditional systems, promotes the accumulation and dissemination of pathogens, reduces plant vigor, and affects the availability of healthy material across successive growing seasons [5,6]. Moreover, ex situ conservation and the proper maintenance of germplasm collections may be compromised when the initial material does not meet phytosanitary standards or when propagation is insufficiently rapid and uniform to sustain conservation and breeding programs [7,8,9]. These limitations highlight the need for strategies that ensure the production of healthy, uniform, and reproducible plants, particularly when aiming to preserve and utilize germplasm of agronomic value.

Given these limitations, in vitro plant tissue culture, particularly micropropagation, represents a well-established tool for the rapid multiplication of elite genotypes, the production of pathogen-free plants, and the year-round availability of plantlets under controlled conditions [10,11]. Furthermore, the standardization of regeneration and multiplication protocols supports the ex situ conservation of native genetic resources, facilitates the safe distribution of plant material, and enables subsequent applications in plant biotechnology and advanced breeding [11,12]. Therefore, for Andean species characterized by vegetative propagation and high genetic diversity, the development of efficient and transferable protocols serves not only productive purposes but also contributes to conservation and sustainable use of these resources [10,13]. In addition, such protocols provide a necessary foundation for future breeding applications in ulluco, including genetic transformation and genome editing [14,15], as efficient in vitro regeneration is a prerequisite for the implementation of these approaches in underexplored crops.

Evidence in ulluco indicates that in vitro plant regeneration from explants is achievable using appropriate combinations of auxins and cytokinins, and that viruses can be eradicated through thermotherapy and in vitro chemotherapy. However, responses vary depending on genotypes and culture conditions [11,13,16]. The efficiency of these responses, measured in terms of multiplication rate, shoot quality, root induction, and acclimatization survival, differs considerably among genotypes and culture environments. This variability limits the transferability of generalized protocols and reduces their practical applicability in local production systems [11,17,18]. Therefore, for Peruvian regional genotypes, combinations of growth regulators and culture conditions for regional genotypes of *Ullucus tuberosus* still require optimization and validation to establish a reproducible protocol for the multiplication and establishment of uniform, healthy, and traceable plant material for both conservation and productive use [19,20].

Therefore, the objective of this study was to develop an efficient and reproducible in vitro micropropagation protocol for a genotype of *Ullucus tuberosus* from the Amazonas region of Peru, integrating shoot multiplication, rooting, and acclimatization stages, with the aim of contributing to the conservation, availability, and sustainable use of this Andean plant genetic resource.

## 2. Results and Discussion

### 2.1. Induction and Proliferation of Shoots

The factorial analysis of cytokinin-containing treatments revealed that the in vitro response of *Ullucus tuberosus* was differentially influenced by cytokinin type, concentration, and their interaction, depending on the variable evaluated (Table 1). The interaction effect was significant for regeneration, number of nodes, and shoot length, indicating that the response to increasing cytokinin concentration differed between BAP and KIN supplemented media. In contrast, shoot number was not significantly affected by cytokinin type, concentration, or their interaction in the factorial analysis. Nevertheless, the overall one-way ANOVA including the control treatment was significant for all variables, confirming that the absence of plant growth regulators contributed to the observed treatment differences. 

**Figure 1:**
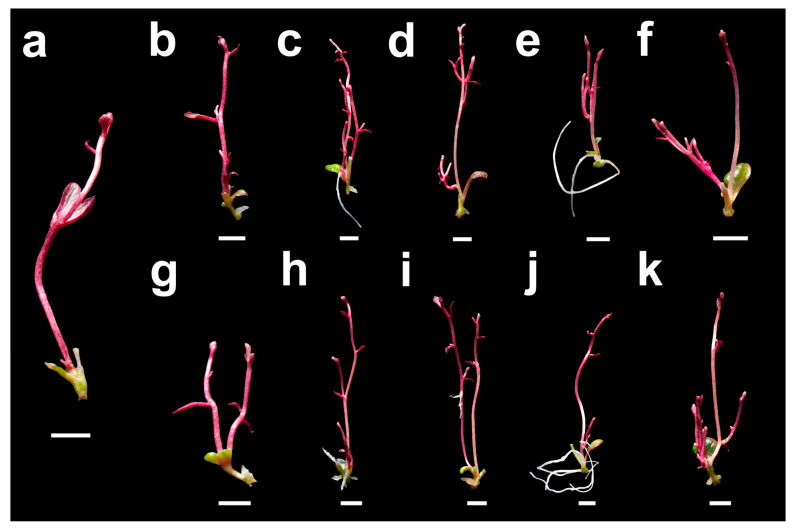
Morphogenic response of *Ullucus tuberosus* explants (accession UT-001) after four weeks of culture on MS medium supplemented with cytokinins. (**a**) Explants cultured on MS medium without growth regulators; (**b**–**f**) explants cultured on MS medium supplemented with BAP at 0.1, 0.2, 0.5, 1.0, and 2.0 mg L^−1^; (**g**–**k**) explants cultured on MS medium supplemented with KIN at 0.1, 0.2, 0.5, 1.0, and 2.0 mg L^−1^. Scale bar = 5 mm (all panels).

*Ullucus tuberosus* explants responded positively to morphogenesis both in MS medium without PGR (control) and in media supplemented with cytokinins; however, the addition of BAP or KIN significantly enhanced and modulated shoot regeneration and vigor (Table 2). The control treatment showed 80.00% regeneration, whereas the highest response was obtained with KIN at 2.0 mg L^−1^, reaching 100.00%, confirming the high effectiveness of this cytokinin in inducing organogenesis in this explant type. Regarding shoot proliferation, cytokinin treatments increased the number of shoots compared with the control (Figure 1), with BAP at 0.2 mg L^−1^ showing the highest value (2.07 ± 0.21), although it was not significantly different from other BAP and KIN concentrations. Similarly, the number of nodes per explant increased markedly in the presence of cytokinins, reaching values close to 4.80. In terms of shoot elongation, KIN at 1.0 mg L^−1^ produced the longest shoots (39.15 ± 0.37 mm), followed by KIN at 0.5 and 2.0 mg L^−1^, with mean lengths of 35.53 and 35.37 mm, respectively.

Our results confirm that the morphogenic performance of *Ullucus tuberosus* during the multiplication phase depends on the type and concentration of cytokinin. This pattern was particularly evident with BAP, which promoted the activation of axillary buds and the establishment of new shoot meristems, whereas KIN shifted the response toward greater shoot elongation and enhanced nodal development, traits particularly valuable for sustaining successive subcultures and obtaining material suitable for rooting. Other studies have reported the combined use of these cytokinins. For example, Jordan et al. [11] demonstrated that combinations of BAP and kinetin enhance organogenesis and multiplication in *U. tuberosus*. These findings are consistent with reports in Andean species, where kinetin has been associated with more orderly growth and greater clonal fidelity during in vitro conservation [12,21,22].

The superior performance of KIN could be attributed to its metabolic dynamics within plant tissues, characterized by a more sustained activity in meristematic regions and a lower tendency to induce excessive shoot compaction, thereby promoting greater internode elongation and increased nodal development [13]. In contrast, BAP tends to maximize initial bud proliferation but may shift the balance toward intense cell division, resulting in more compact shoots if not followed by an elongation phase [23,24].

Compared with other studies on the in vitro propagation of ulluco, the highest average number of shoots per explant in our study was obtained with BAP at 0.2 mg L^−1^; however, this value was lower than that reported by Jordan et al. [11]. These authors cultured polynodal sections (containing three to four axillary buds) and obtained up to 18.3 shoots per explant using liquid MS medium supplemented with 0.1 mg L^−1^ of BAP, 0.1 mg L^−1^ of gibberellic acid (GA_3_), and 0.01 mg L^−1^ of NAA. Regarding shoot length, Jordan et al. [11] reported an average of 26.3 mm using liquid MS medium supplemented with 1.0 mg L^−1^ of thidiazuron, 1.0 mg L^−1^ of GA_3_, and 0.3 mg L^−1^ of NAA, which was lower than the maximum value obtained in our study (39.15 mm) with MS medium supplemented with 1.0 mg L^−1^ of KIN. Nevertheless, our shoot length values were lower than those reported by Hammond et al. [12], who achieved an average of 56.7 mm using half-strength MS medium supplemented with 30.0 g L^−1^ of mannitol.

From an applied perspective, our findings suggest that micropropagation can serve as a valuable tool for the conservation and sustainable use of Andean crops by facilitating the rapid production of uniform planting material under controlled conditions [25]. However, genetic fidelity and sanitary quality should be verified for each protocol, particularly when plant growth regulators are used over successive subcultures [24,26]. In addition, the genotype-dependent nature of in vitro responses, widely reported in tissue culture studies, highlights the need to optimize regeneration protocols for regional germplasm rather than indiscriminately extrapolating hormone combinations across accessions or provenances [3,6,27].

### 2.2. Root Induction

The factorial analysis of auxin-containing treatments showed that the in vitro rooting response of *Ullucus tuberosus* was significantly influenced by auxin concentration and by its interaction with auxin type for all evaluated variables (Table 3). Auxin type alone did not significantly influence rooting percentage or roots number, whereas it had a significant effect on root length. The significant interaction for all variables indicates that the response to increasing auxin concentration differed between IBA- and NAA-supplemented media. Moreover, the one-way ANOVA including the control treatment was significant for all rooting traits, confirming overall differences among treatments when the absence of plant growth regulators was also considered.

**Figure 2:**
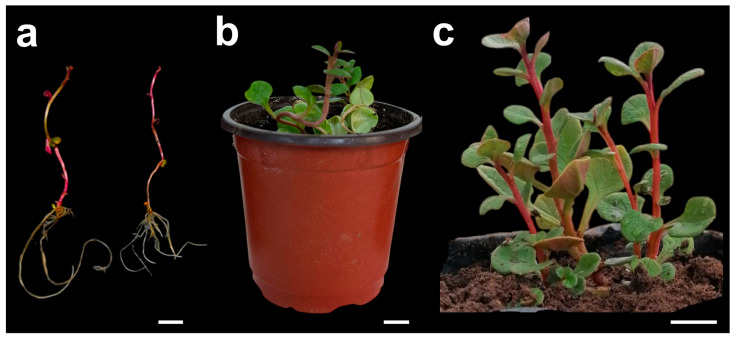
In vitro rooting and acclimatization of *Ullucus tuberosus* (accession UT–001). (**a**) In vitro-regenerated plantlet grown on MS rooting medium supplemented with auxins, showing adventitious root formation after eight weeks (scale bar = 1 cm); (**b**) plantlet acclimatized for four weeks in a pot containing sterile agricultural substrate and humus (scale bar = 1 cm); (**c**) plantlet acclimatized for ten weeks in a bag containing sterile agricultural substrate and humus, showing vigorous growth (scale bar = 3 cm).

Rooting of *Ullucus tuberosus* shoots was observed both in MS medium without PGRs (control) and on media supplemented with auxins; however, the type and, especially, the concentration of auxin significantly influenced the response (Table 4). The control treatment achieved 86.67% rooting, with 3.53 ± 0.22 roots per shoot and an average length of 31.99 ± 1.25 mm, demonstrating an inherent capacity for in vitro rooting. In the presence of IBA, root induction followed a dose-dependent pattern. At 0.1 mg L^−1^, the highest number of roots (4.67 ± 0.21) was obtained, significantly exceeding the other IBA concentrations. However, these roots were shorter (23.27 ± 0.36 mm) than those of the control, suggesting that low concentrations of IBA primarily promote root initiation rather than elongation. As the IBA concentration increased (0.2–2.0 mg L^−1^), the rooting percentage decreased (73.33–60.00%) and both the number and length of roots were markedly reduced, reaching minimum values at 2.0 mg L^−1^ (1.47 ± 0.11 roots; 5.55 ± 0.29 mm), which is consistent with an inhibitory effect at high concentrations. In contrast, NAA at 0.1 mg L^−1^ produced the most favorable overall response, achieving 100% regeneration and the greatest root elongation (41.93 ± 0.59 mm), while maintaining a number of roots comparable to the control. These results indicate that NAA at 0.1 mg L^−1^ optimizes both rooting percentage and root elongation, whereas IBA at 0.1 mg L^−1^ maximizes root number, albeit with a shorter root system (Figure 2a).

In vitro rooting of *U. tuberosus* from shoot explants demonstrated that auxins play an essential role in the development and differentiation of adventitious roots by regulating cell division, polar transport, and lateral root formation processes [28]. Previous studies have shown that exogenous application of IBA and NAA promotes root elongation, increases root number, and enhances root biomass in various plant species [13,20]. These findings are consistent with the results of the present study, in which low concentrations of IBA (0.1 mg L^−1^) and NAA (0.1 mg L^−1^) promoted a more efficient rooting response, confirming the positive effect of these auxins on in vitro-propagated species.

Compared to other studies on the in vitro rooting of ulluco, our most effective treatment (0.1 mg L^−1^ IBA) produced an average of 4.67 roots per explant, exceeding the results reported by Jordan et al. [11], who obtained a mean of 2.0 roots per explant using liquid MS medium supplemented with 1.0 mg L^−1^ of thidiazuron, 1.0 mg L^−1^ of GA3, and 0.3 mg L^−1^ of NAA. However, our results were lower than those reported by Hammond et al. [12], who achieved an average of 6.38 roots per explant using half-strength MS medium supplemented with 30.0 g L^−1^ of mannitol.

The use of IBA as the primary auxin for rooting has been widely reported in root and tuber crops, such as *Solanum tuberosum* and *Oxalis tuberosa*, where concentrations between 1.0–1.5 mg L^−1^ promote the formation of thick and functional adventitious roots [10,21,29]. This effect has been attributed to the greater stability of IBA compared with NAA, allowing a gradual and sustained release that stimulates cell differentiation at the base region of the stem [20].

### 2.3. Acclimatization of Plants Regenerated In Vitro

In vitro-rooted plants of *Ullucus tuberosus* exhibited a 100% survival rate by the eighth week of acclimatization (Figure 2b). Subsequently, they were transferred to bags containing the same substrate, where they continued to grow vigorously, as observed at ten weeks (Figure 2c). None of the plantlets showed signs of physiological stress or pathogen infection during the acclimatization period, and active, uniform growth was consistently recorded. This performance confirms that the quality of the root system developed in vitro is a key determinant of ex vitro establishment success. Furthermore, maintaining a controlled environment during the initial acclimatization phase mitigates transplant shock associated with the change in atmospheric conditions, thereby facilitating the transition to photoautotrophic growth [30,31].

Other studies on ulluco have also reported successful acclimatization. For example, Hammond et al. [12] achieved 100% survival using a sterile mixture of garden substrate and perlite. Similar outcomes have been documented in other crops, highlighting that well-developed, elongated roots, together with adequate control of humidity and light, significantly enhance plant establishment during acclimatization [21,32,33,34].

Acclimatization represents a critical stage in the micropropagation process, as it determines the ultimate success of the ex vitro establishment of regenerated plants [29,35,36]. During this phase, plantlets must adapt to environmental conditions that differ markedly from those of in vitro culture, including the loss of a sterile environment, reduced relative humidity, and increased irradiance [37,38]. These changes may trigger abiotic stress responses (for example, dehydration, light stress, and osmotic imbalance) as well as biotic stress responses (for example, microbial infection), potentially compromising plantlet viability and early growth of the plantlets [39]. Therefore, careful management of humidity, temperature, ventilation, and substrate conditions is essential to ensure a successful transition [40].

The substrate used during acclimatization directly influences the survival, development, and vigor of plantlets obtained through in vitro culture [38]. A porous and well-aerated substrate with adequate water-holding capacity, such as a mixture of agricultural soil and humus (2:1), promotes gas exchange and supports the formation of a strong and functional root system [41]. In addition, humus contributes to beneficial microbial colonization and enhances nutrient availability, thereby facilitating plantlet adaptation and early growth [42,43].

In this study, *U. tuberosus* plantlets exhibited excellent foliar and root development following transfer to the substrate, with active growth and no symptoms of wilting, chlorosis, or necrosis, thereby demonstrating the effectiveness of the rooting and acclimatization protocol. These results indicate that the *U. tuberosus* genotype from Amazonas possesses a high capacity for ex vitro adaptation, supporting its potential for large-scale conservation and propagation programs [3].

The implications of these results are significant for the preservation of Andean germplasm and the genetic improvement of ulluco, as a successful and reproducible micropropagation protocol ensures the availability of healthy, vigorous, and phenotypically uniform plants [44]. Future research could expand this approach by evaluating additional genotypes from different regions of Peru and further optimizing environmental conditions during acclimatization [3].

## 3. Materials and Methods

### 3.1. Initiation of Aseptic Cultivation

**Figure 3:**
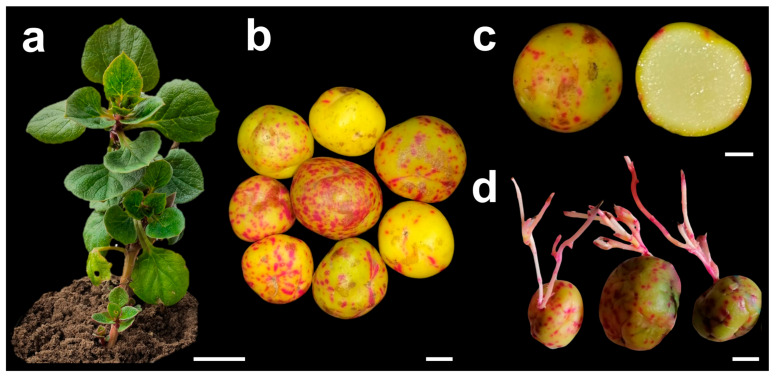
Plant material from accession UT–001 of *Ullucus tuberosus* used for in vitro establishment: (**a**) cultivated donor plant (scale bar = 5 cm); (**b**) tubers harvested from cultivated plants (scale bar = 1 cm); (**c**) cross-section of tubers showing internal coloration (scale bar = 1 cm); (**d**) tubers approximately 30 days old kept at room temperature, showing shoots used as a source of explants (scale bar = 1 cm).

Ulluco tubers from accession UT–001 were collected in the district of Lamud (2330 m a.s.l.), province of Luya, Amazonas region, Peru. The tubers were rounded, yellow with pink spots, and smooth with a floury texture. Healthy tubers without visible mechanical damage or disease symptoms were selected. Tubers were established in pots (20 cm in height and 15 cm in diameter) containing a sterile mixture of agricultural substrate at the Laboratorio de Biología Molecular de Plantas (LBMP) of the Universidad Nacional Toribio Rodríguez de Mendoza (UNTRM), where they were grown for plant development (Figure 3a) and tuber production (Figure 3b,c). The tubers were maintained at room temperature (20–22 °C) in the dark and under ambient relative humidity, for approximately 30 days to induce sprouting. The emerging shoots were used as a source of explants (Figure 3d).

The sprouts were washed with neutral detergent (Ayudín, Lima, Peru) at 1% (*v*/*v*) for 15 min under running tap water and then surface-sterilized with 70% ethanol for 1 min, followed by 1% sodium hypochlorite (NaClO) (PhytoTech Labs, Lenexa, KS, USA) for 10 min with gentle agitation. Subsequently, they were rinsed three times with sterile distilled water to remove any residual disinfectant. Under aseptic conditions, nodal segments approximately 1 cm in length were excised and cultured on half-strength MS medium [45] (PhytoTech Labs, Lenexa, KS, USA) supplemented with 1.5% (*w*/*v*) sucrose (PhytoTech Labs, Lenexa, KS, USA) and 0.7% (*w*/*v*) agar (PhytoTech Labs, Lenexa, KS, USA), adjusted to pH 5.8 [21]. Cultures were incubated in a growth chamber (ICH750L Memmert, Schwabach, Germany) at 24 ± 1 °C under a light intensity of 80 μmol m^−2^ s^−1^ with a 16/8 h light/dark photoperiod.

### 3.2. Induction and Proliferation of Shoots

Shoots approximately 10 cm in length were obtained from previously established nodal segments (30 days old). The nodal segments were excised to approximately 1 cm and cultured on MS medium without plant growth regulators (PGRs) or on MS medium supplemented with independent gradients of 6-benzylaminopurine (BAP) (PhytoTech Labs, Lenexa, KS, USA) or kinetin (KIN) (PhytoTech Labs, Lenexa, KS, USA) at concentrations of 0.1, 0.2, 0.5, 1.0, and 2.0 mg L^−1^. All media were prepared with 3% (*w*/*v*) sucrose and 0.7% (*w*/*v*) agar and adjusted to pH 5.8 prior to autoclaving. Cultures were incubated at 24 ± 1 °C under a light intensity of 80 μmol m^−2^ s^−1^ with a 16/8 h light/dark photoperiod. After four weeks, the regeneration percentage, number of shoots per explant, number of nodes per explant, and shoot length were evaluated.

### 3.3. Root Induction

Healthy explants (1.0 cm in length) obtained from regenerated shoots of the previous phase were excised and cultured on rooting media consisting of MS medium without PGRs or supplemented with independent gradients of indole-3-butyric acid (IBA) (PhytoTech Labs, Lenexa, KS, USA) or 1-naphthaleneacetic acid (NAA) (PhytoTech Labs, Lenexa, KS, USA) at concentrations of 0.1, 0.2, 0.5, 1.0, and 2.0 mg L^−1^. All media were supplemented with 3% (*w*/*v*) sucrose, 0.7% (*w*/*v*) agar, and adjusted to pH 5.8 prior to autoclaving. Cultures were incubated at 25 ± 1 °C under a light intensity of 80 μmol m^−2^ s^−1^ with a 16/8 h light/dark photoperiod. After four weeks, the rooting percentage, number of roots per explant, and root length were evaluated.

### 3.4. Acclimatization of Plants Regenerated In Vitro

In vitro-rooted ulluco plantlets were removed from their culture vessels and gently rinsed with distilled water to eliminate any adhering medium. The plantlets were transplanted into small pots (8 cm in height and 8 cm in diameter) containing a sterile mixture of agricultural substrate and humus (2:1). They were maintained in a growth chamber at 25 ± 2 °C under a light intensity of 80 μmolm^−2^ s^−1^ with a 16/8 h light/dark photoperiod and 60% relative humidity, without an additional high-humidity enclosure. Plants were irrigated every two days. The survival rate was evaluated eight weeks after transplantation.

### 3.5. Experimental Design and Statistical Analysis

The experiments were arranged in a completely randomized design (CRD). For each treatment, three explants were cultured per replicate, with 15 replicates per treatment, resulting in a total of 45 explants per experiment. The assumptions of normality and homogeneity of variances were assessed using the Shapiro–Wilk test [46] and Levene’s test [47], respectively. After verifying these assumptions, the data were subjected to one-way and two-way analysis of variance (ANOVA). A factorial ANOVA was used to test the effects of regulator type, concentration, and their interaction (F-test), and the mean comparisons were performed using Tukey’s HSD test (α = 0.05) [48]. Statistical analyses were conducted using R software (version 4.5.1 for Windows) [49], with the car [50] and agricolae [51] packages.

## 4. Conclusions

This study establishes a reproducible protocol for the in vitro micropropagation of the native *Ullucus tuberosus* accession UT–001 from Amazonas, Peru. During the multiplication phase, explants exhibited a high basal morphogenic capacity on MS medium, and cytokinin supplementation significantly enhanced and modulated the response. In particular, BAP at 0.2 mg L^−1^ maximized shoot proliferation (2.07 shoots per explant) and consistently increased the number of nodes, indicating a favorable profile for successive subcultures and clonal multiplication. In the rooting phase, IBA at 0.1 mg L^−1^ produced the highest number of roots (4.67). During the ex vitro phase, rooted plantlets achieved 100% survival after eight weeks of acclimatization and maintained uniform growth through the tenth week. Overall, this protocol represents a reliable approach for the rapid propagation of uniform planting material and may support ex situ conservation and future biotechnological applications in ulluco. However, because the present study was conducted using a single native accession, the extent to which this protocol is genotype-dependent remains unknown. Therefore, future research should evaluate its applicability across a broader range of ulluco accessions from different geographic origins under the same culture conditions.

## Figures and Tables

**Table 1:** Εffect of the experimental factors, cytokinin type (BAP and KIN) and concentration (0.1, 0.2, 0.5, 1.0, 2.0 mg L^−1^) on the in vitro response of *Ullucus tuberosus*.

F-Test	Regeneration (%)	Shoots Number	Nodes Number	Shoot Length (mm)
*F*_PGR_	ns	ns	**	***
*F _concentration_*	***	ns	**	***
*F _PGR×concentration_*	***	ns	***	***
*F* _one-way ANOVA_	***	***	***	***

ns or **, ***: non-significant at *p* ≤ 0.05 or significant at *p* ≤ 0.01, *p* ≤ 0.001. Control treatment (MS medium without PGRs) was not included in the two-way ANOVA and was considered only in the one-way ANOVA.

**Table 2:** Effect of BAP and KIN on shoot induction from nodal explants of *Ullucus tuberosus*.

PGR	Concentration (mg L^−1^)	Regeneration (%)	Shoots Number	Nodes Number	Shoot Length (mm)
Control	0.0	80.00 ^d^	1.07 ± 0.07 ^b^	2.33 ± 0.21 ^d^	17.11 ± 0.39 ^e^
BAP	0.1	80.00 ^d^	1.47 ± 0.13 ^ab^	4.73 ± 0.18 ^a^	15.17 ± 0.59 ^e^
	0.2	93.33 ^b^	2.07 ± 0.21 ^a^	4.13 ± 0.19 ^ab^	24.17 ± 0.66 ^d^
	0.5	86.67 ^c^	1.60 ± 0.13 ^ab^	4.67 ± 0.29 ^a^	29.02 ± 1.04 ^c^
	1.0	80.00 ^d^	1.80 ± 0.18 ^a^	4.07 ± 0.21 ^ab^	20.86 ± 0.51 ^d^
	2.0	73.33 ^e^	1.80 ± 0.11 ^a^	3.60 ± 0.13 ^bc^	21.22 ± 0.48 ^d^
KIN	0.1	80.00 ^e^	1.53 ± 0.13 ^ab^	3.13 ± 0.22 ^cd^	17.04 ± 0.45 ^e^
	0.2	86.67 ^c^	1.73 ± 0.12 ^a^	3.07 ± 0.21 ^cd^	23.89 ± 0.52 ^d^
	0.5	86.67 ^c^	1.73 ± 0.12 ^a^	4.07 ± 0.19 ^ab^	35.53 ± 1.38 ^b^
	1.0	73.33 ^e^	1.47 ± 0.13 ^ab^	4.47 ± 0.13 ^ab^	39.15 ± 0.37 ^a^
	2.0	100.00 ^a^	1.73 ± 0.18 ^a^	4.80 ± 0.22 ^a^	35.37 ± 0.88 ^b^

Means ± SE followed by different letters are significantly different according to Tukey’s test (*p* ≤ 0.05).

**Table 3:** Εffect of the experimental factors, auxin type (IBA and NAA) and concentration (0.1, 0.2, 0.5, 1.0, 2.0 mg L^−1^) on the in vitro response of *Ullucus tuberosus*.

F-Test	Rooting (%)	Roots Number	Root Length (mm)
*F* _PGR_	ns	ns	***
*F _concentration_*	***	***	***
*F _PGR×conc._*	***	***	***
*F* _one-way ANOVA_	***	***	***

ns or ***: non-significant at *p* ≤ 0.05 or significant at *p* ≤ 0.001. Control treatment (MS medium without PGRs) was not included in the two-way ANOVA and was considered only in the one-way ANOVA.

**Table 4:** Effect of NAA and IBA on root induction from shoot explants of *Ullucus tuberosus*.

PGR	Concentration (mg L^−1^)	Rooting (%)	Roots Number	Root Length (mm)
Control	0.0	86.67 ^b^	3.53 ± 0.22 ^b^	31.99 ± 1.25 ^b^
IBA	0.1	86.67 ^b^	4.67 ± 0.21 ^a^	23.27 ± 0.36 ^cd^
	0.2	73.33 ^d^	2.93 ± 0.21 ^c^	9.79 ± 0.46 ^ef^
	0.5	73.33 ^d^	2.53 ± 0.22 ^d^	8.48 ± 0.41 ^f^
	1.0	73.33 ^d^	2.33 ± 0.13 ^d^	7.46 ± 0.30 ^fg^
	2.0	60.00 ^e^	1.47 ± 0.11 ^f^	5.55 ± 0.29 ^g^
NAA	0.1	100.00 ^a^	3.47 ± 0.26 ^b^	41.93 ± 0.59 ^a^
	0.2	73.33 ^d^	2.27 ± 0.27 ^d^	22.40 ± 0.44 ^d^
	0.5	80.00 ^c^	3.60 ± 0.19 ^b^	25.56 ± 0.82 ^c^
	1.0	60.00 ^e^	2.40 ± 0.19 ^d^	11.65 ± 0.49 ^e^
	2.0	53.33 ^f^	1.60 ± 0.11 ^e^	5.44 ± 0.21 ^g^

Means ± SE followed by different letters are significantly different according to Tukey’s test (*p* ≤ 0.05).

## Data Availability

The datasets used and analyzed in this study are available from the corresponding author upon reasonable request.
